# Bovine Brain: An *in vitro* Translational Model in Developmental Neuroscience and Neurodegenerative Research

**DOI:** 10.3389/fped.2014.00074

**Published:** 2014-07-10

**Authors:** Antonella Peruffo, Bruno Cozzi

**Affiliations:** ^1^Department of Comparative Biomedicine and Food Science, University of Padova, Padova, Italy

**Keywords:** brain, neurodegenerative research, translational model, fetal alcohol syndrome, *in vitro* model

## Abstract

Animal models provide convenient and clinically relevant tools in the research on neurodegenerative diseases. Studies on developmental disorders extensively rely on the use of laboratory rodents. The present mini-review proposes an alternative translational model based on the use of fetal bovine brain tissue. The bovine (*Bos taurus)* possesses a large and highly gyrencephalic brain and the long gestation period (41 weeks) is comparable to human pregnancy (38–40 weeks). Primary cultures obtained from fetal bovine brain constitute a validated *in vitro* model that allows examinations of neurons and/or glial cells under controlled and reproducible conditions. Physiological processes can be also studied on cultured bovine neural cells incubated with specific substrates or by electrically coupled electrolyte-oxide-semiconductor capacitors that permit direct recording from neuronal cells. Bovine neural cells and specific *in vitro* cell culture could be an alternative in comparative neuroscience and in neurodegenerative research, useful for studying development of normal and altered circuitry in a long gestation mammalian species. Use of bovine tissues would promote a substantial reduction in the use of laboratory animals.

## Animal Models in Research on Human Neurodegenerative Disorders: A Brief Overview

Neurodegenerative diseases are a heterogeneous group of disorders characterized by impairment of neuronal structure and function, and are generally accompanied by neuronal loss.

There is a growing interest in the development of novel animal models (1) and transgenic systems (2), to understand the cellular and molecular basis of human neurodegenerative disorders. Translational medicine is constantly evolving and significant progress has been recently made through the improvement of well-established models and the development of original paradigms (3).

In Table 1, we report a selection of reviews focusing on model organisms used in experimental research on human neurodegenerative disorders. Rodents (mice and rats) remain the most widely used species for modeling human neurodegenerative syndromes (4–6). Additional species (cats, dogs, and primates) are used in Parkinson’s (7) and Huntington’s disease studies (8). Pigs, sheep, and primates are employed besides rodents to study the fetal alcohol syndrome (9).

**Table 1:** **Animal models of human neurodegenerative disorders**.

Disorder	Species	Review articles
Alzheimer’s disease	Rodents	Gotz and Ittner (5)
Parkinson’s disease	Rodents, cats, dogs, and primates	Betarbet et al. (7)
Depression	Rodents	Yan et al. (6)
Schizophrenia	Rodents	Mouri et al. (4)
Huntington’s disease	Primates	Aron Badin and Hantraye (8)
Fetal alcohol syndrome	Rodents, pig, sheep, and primates	Cudd (9)

While rodents are strategic models because of their ease of management, fast reproduction, and low maintenance cost; larger mammals may also be useful because their more complex anatomy and physiology make them more directly comparable to humans in some respects (10). New translational models are also relevant to understand the response to treatment of specific neurodegenerative processes, and essential to better comprehend the natural history of a given disease (1). The goal of this mini-review is to summarize a few issues on the use of the bovine as an alternative experimental model in neurodegenerative research, including the fetal alcohol spectrum disorders (FASDs).

## Bovine: A Large Mammal with a Large Brain

The bovine species *Bos taurus* is a widespread domestic mammal, raised worldwide for meat and milk production. The bovine possesses a relatively large (approximately 600 g), highly gyrencephalic brain, in comparison to the smooth-surfaced brain of laboratory rodents (11). Furthermore, the CNS of bovine is easily available in large quantities at the slaughterhouse wherever this species is present.

The long gestation period of the bovine (41 weeks) is comparable to human pregnancy (38–40 weeks). During the last decade, our laboratory used this species to study the role of sexual steroids in the regulation of brain differentiation and the expression of cytochrome P450 aromatase, the key enzyme of estrogen biosynthesis (12), in relation to specific estrogen receptor subtypes (ERs). We quantified the expression profiles and neural localization of aromatase P450 and estrogen receptors α and β during consecutive developmental stages in fetal bovine hypothalamus and cerebral cortex (13, 14). Quantitative data analysis on expression patterns of both ERs in different bovine fetal brain regions indicates a strong reciprocal correlation during pregnancy and an increase in the last stage of gestation (14). Our data highlighted that the early second quarter of the gestation (fourth month) is the critical period for hypothalamic differentiation in bovine ontogenesis. This is an important difference with respect to short gestation species (rat and mouse), where aromatase activity peaks around delivery. In fact, in long gestation species like the bovine and human, the critical period for sexual differentiation occurs in earlier gestation phases (13, 15, 16). It must be noted that the bovine CNS matures comparatively early during pregnancy, since newborn calves must be immediately able to stand, move, and relate to the external world. In contrast, in short gestation species brain differentiation and development continue throughout the perinatal period and afterwards (13, 15, 17). This latter feature only apparently resembles human neoteny, as maturation of the CNS in our species continues for decades but the general organization of the brain is well-established by mid-gestation. The use of fetal bovine brain tissues in experimental medicine may become a valid alternative to laboratory mammals in all those instances in which rodent physiology widely differs from human physiology (see Table 2).

**Table 2:** **Use of bovine tissues for the study of altered sexual differentiation and neurodegenerative research**.

Neurodegenerative disorder	Review
Twin–twin transfusion syndrome	Padula (18)
Batten disease	Weber and Pearce (19)
Neuronal ceroid lipofuscinosis	Bond et al. (20)
Prion diseases	Imran and Mahmood (21)

An important contribution in remodeling and reshaping of fetal CNS during neural differentiation is performed by the voltage operated calcium channels (VOCCs) that influence cell migration, neuronal sprouting, synaptogenesis, and neurotransmitter release (22–25). The VOCCs are crucial for brain function, and their incorrect expression and/or dysfunction gives rise to a variety of neurological disorders, including pathological pain, epilepsy, migraine, and ataxia (26).

The VOCCs are involved in the maintenance of intracellular Ca^2+^ homeostasis. An increase in intracellular Ca^2+^ triggers a wide range of intracellular processes, such as activation of calcium-dependent enzymes, gene transcription, and neurotransmitter release (27, 28). The properties of the VOCCs are largely conferred by their pore-forming α1-subunits. An impairment of calcium signals is also observed in experimental models of FASD (29). The bovine fetal hypothalamus is a potential tool to evaluate the contribution that VOCCs make to brain development. A recent article assessed the expression of a P/Q and L-type VOCCs by real time RT-PCR, and quantified α1A and α1D subunit expression in the bovine hypothalamus, at various stages of development (30). Data showed that the profile expression of these subunits peaks during the last period of the gestation in the male hypothalamus, in which the expression of α1A and α1D shows higher values than in females. In females, the expression profiles of both genes were constant throughout development (30).

The high expression of α1A and α1D during development suggests that the presence of an increased density of P/Q and L-type VOCCs, which may be involved in the process of sexual differentiation during development, an hypothesis also supported by other studies (22). Sex differences in the levels of L and P/Q channel expression may be a part of the mechanism leading to the onset of activities that control differentiation in young CNS neurons. Moreover, their activity may be crucial for physiological responses of neuronal populations, starting from the second half of the pregnancy when the architecture of bovine hypothalamus is defined and networks start to develop.

## Bovine Brain: A Translational Model for Altered Sexual Differentiation of the Brain and Neurodegenerative Research

Large animals are more similar to humans in relation to brain size and lifespan and could be therefore essential to investigate complex patho-physiological mechanisms relating to neurodegenerative diseases and infectious neuropathologies (31).

The bovine freemartin syndrome, the most frequent form of intersexuality found in cattle, may represent a useful model in which to study the human condition called twin–twin transfusion syndrome. Freemartins develop when vascular connections are established between the placentas of developing heterosexual twin fetuses, and the result is masculinization of the female reproductive tract to varying degrees due to the high circulating levels of testosterone (18). These natural born intersex calves could represent an ideal model to study sexual patho-physiological evolution of sexual brain differentiation in mammals. These bovine pseudo-hermaphrodite females are a common instance in bovine twin pregnancies (involving one male and one female fetus) and can also be artificially induced.

In humans, twins originating from a single placenta form vascular anastomoses, which may lead to unequal sharing of blood supply and ultimately the impaired development or death of one or both fetuses (32). The infrequent nature of the condition makes comparison of treatment options difficult and the bovine freemartin may represent an animal system in which to study and compare treatments. It is worth noting here that recent findings suggest the possibility that early alcohol exposure may have steroid-mediated sexually dimorphic effects on serotoninergic neurons (33).

A recent review (34) examined the use of non-laboratory or large animal models for neuronal ceroid lipofuscinoses (NCL; Batten disease), a group of fatal progressive neurodegenerative diseases predominantly affecting children. Data from the literature confirm that natural cases of NCL occur in a large variety of species including the bovine (19, 20). Research in prion pathology, the transmissible neurodegenerative conditions affecting human and a wide range of animal species, lead to an increased awareness of the need to use large animal models such as the bovine, in addition to conventional laboratory animals (21).

## *In vitro* Tool for Neurodegenerative Studies

*In vitro* models provide important insights into the pathogenesis of neurodegenerative disorders and represent an interesting approach for the screening of potential pharmacological agents (35, 36). To obtain scientifically valid research, experimental conditions must be strictly controlled: this often involves manipulating one single variable at a time while keeping the others constant, and then observing the consequences of that single specific change. To this effect, primary cultures from fetal bovine hypothalamus and cerebral cortex may be standardized to obtain a reliable and reproducible model.

A number of studies have validated *in vitro* models based on neural primary cultures obtained from fetal bovine hypothalamus, cerebral cortex, and cerebellum, allowing examinations of neurons and/or glial cells under controlled and reproducible conditions. Cell cultures obtained from frozen–thawed bovine fetal tissues are comparable to cultures derived from fresh fragments of cortex and hypothalamus of the same animal, showing similar growth profiles (37, 38). Bovine cultures from the hypothalamus and frontal cortex retain also *in vitro* the ability to express and synthesize the enzyme aromataseP450Arom and the α- and β-estrogen receptors (17). These data are in agreement with data observed in others species, such as mice, rats, and avian species (39–41). Bovine neurons *in vitro* maintain the ability to generate action potentials. Electrolyte-oxide-semiconductor capacitors (EOSCs), a class of microtransducers for extracellular electrical stimulation, may be employed to activate voltage-dependent sodium channels at the neuronal soma, resulting in a versatile complement for the investigation of Ca^2+^ signaling (42).

Bovine cerebellum-derived endothelial cell lines are useful to monitor Ca^2+^ oscillations in the main intracellular compartments including the cytosol, the endoplasmic reticulum, and the mitochondria. Mitochondrial Ca^2+^ uptake significantly decreased after 48-h exposure to estradiol, whereas cytosolic and endoplasmic reticulum responses were unaffected. The permeability transition pore (PTP) may be involved in the mechanism of action and influences energy metabolism and cell viability. Treating cells with cyclosporine A (CsA), which binds to the matrix chaperone cyclophilin-D and regulates PTP opening, reversed the effects of a 48-h treatment with estradiol, thus suggesting a possible transcriptional modulation of proteins involved in the mitochondrial permeability transition process (43).

Fetal alcohol spectrum disorder has also been considered a neurodegenerative disease [see Ref. (44)] with an interesting mechanism involving glutamate receptors and excessive activation of GABA(A) receptors and consequent apoptotic neurodegeneration in the developing rat forebrain. This process could be replicated in bovine primary cultures by defining the proper synaptogenetic phase. Since vulnerability to ethanol exposure coincides with the period of synaptogenesis, which in humans starts from the sixth month of gestation (44), bovine fetal tissues could represent a standardized model and a dynamic system to study molecular mechanisms and physiological process at the cellular level, and potentially practical also for drug discovery.

## Implications for Animal Welfare

The use of experimental animals in biomedical research follows precise national regulations that are increasingly based on the three “Rs” principle (replacement, reduction, and refinement). Investigations on neurodegenerative disorders extensively use laboratory rodents, and reluctance to consider alternative species may derive from a cultural bias.

Our review proposes the use of fetal and adult bovine brain tissue as a potential alternative translational model. Bovine neural tissues employed for experimental studies have the further advantage to be easily obtained in large quantities from slaughterhouses, allowing a considerable reduction in the sacrifice of laboratory animals. Fetal tissues are also widely available, due to the frequent accidental slaughtering of undiagnosed pregnant cows.

A fundamental goal of the Animal Welfare Act is the minimization of animal pain and distress by use of alternative methods. We considered this ethical point of view as the initial criteria of the present mini-review, promoting the development and validation of this new and alternative translational model. In this sense, the use of brain slices is a recognized tool in neurodegenerative investigations (45, 46).

## Conclusion

In this view, the bovine neural cells and specifically the *in vitro* cell cultures could be an alternative of interest in developmental neuroscience and consequently a potential tool for studying the pathophysiology of altered circuitry linked to fetal alcohol exposure during pregnancy (47) in a dynamic system and under standard conditions. Bovine tissues may represent also a novel resource for the study of neurodegenerative disorders.

## Conflict of Interest Statement

The authors declare that the research was conducted in the absence of any commercial or financial relationships that could be construed as a potential conflict of interest.
